# Numerical Study of Wear Characteristics of Vertical Shaft Planetary Mixer Blades

**DOI:** 10.3390/ma18133137

**Published:** 2025-07-02

**Authors:** Shoubo Jiang, Hongwei Zhang, Qingliang Zeng, Qian Du, Xiaopeng Liu

**Affiliations:** College of Mechanical and Electronic Engineering, Shandong University of Science and Technology, Qingdao 266590, China; jiangshoubo@sdust.edu.cn (S.J.); 202383050136@sdust.edu.cn (H.Z.); 202483050090@sdust.edu.cn (Q.D.); xiaopengl@sdust.edu.cn (X.L.)

**Keywords:** mixer blades, wear characteristics, numerical simulation, abrasive wear

## Abstract

The wear failure of vertical shaft planetary mixer blades under complex working conditions directly affects the quality and productivity of concrete. Given that it is time-consuming and labor-intensive to obtain the wear characteristics of mixer blades by experimental methods, this study used numerical simulation to analyze the effects of different factors on the wear characteristics of mixer blades based on the Hertz–Mindlin with JKR cohesive contact model and the Archard wear model. The results of this study show that under the influence of different factors, the blade is subjected to tangential cumulative contact energy and contact force is significantly larger than that in the normal direction, the wear of the blade is judged to be the form of abrasive wear accompanied by impacts, and the wear on the outer middle and lower edge regions of the blade is the most serious. Specifically, for every 5 rpm increase in mixing speed, the blade wear rate increases by 24.14% on average; for every 5° increase in blade angle, the blade wear rate decreases by 2.9% on average; for every 10% increase in the mass ratio of stone aggregate, the blade wear rate increases by 5.95% on average; conical aggregates have the most serious effect on blade wear, while spherical aggregates have the most minor effect. This study provides the theoretical basis and numerical support for understanding the reasons for blade wear loss and enhancing the service life of mixer blades.

## 1. Introduction

Concrete, as the main material for infrastructure construction, occupies a crucial position in modern civil engineering [1,2,3]. The structural parameters and mixing process of the mixer have a significant impact on the quality of concrete [4,5,6,7]. Among them, the mixer blade, as the core component of the mixer, may fail when the wear reaches a certain degree, which in turn affects the quality and productivity of the concrete material. Therefore, an in-depth study of the wear failure mode of mixer blades is crucial for revealing the wear mechanism and identifying the cause of failure.

In many industrial applications, the wear resistance of materials has been one of the key factors affecting the performance and service life of equipment. In recent years, some researchers have analyzed the wear characteristics of horizontal mixer blades by experimental methods, determined the effect of blade inclination angle on the wear pattern, and analyzed the wear resistance of blades made of different materials [8,9,10]. However, with the continuous advancement of new technologies and the increasing complexity of material service environments, traditional experimental research methods face many challenges in terms of cost and time. Therefore, researchers have developed a variety of techniques to improve the wear resistance of materials, including surface coating techniques, heat treatment techniques, and the development of composite materials. For example, using response surface methodology, it was found that applying a NiP/TiN composite coating on the surface of aluminum alloy can significantly improve its wear resistance [11]. The wear process was accelerated by using soft 3D-printed impeller materials, which enabled rapid assessment of wear patterns and wear rates [12]. These technological developments not only increase the service life of the material but also provide more cost-effective solutions for industrial applications.

Nowadays, numerical simulation techniques are becoming an important tool to study the mixing and wear behavior of materials due to their efficiency and flexibility [13,14,15], among which discrete element methods are widely used [16,17,18]. In many industrial applications, the motion of solid particles is closely related to numerous processes, and the use of discrete element methods to analyze particle behavior is particularly important [19,20]. Many scholars have utilized this method to conduct relevant studies on concrete mixing equipment. Researchers have numerically analyzed the blade wear, energy consumption, and mixing quality of different types of mixers, respectively, and found that factors such as blade angle and mixing speed have a significant effect on the performance and wear resistance of mixers [21,22,23,24]. Separately, the method was utilized to confirm that the wear resistance of mixing blades can be improved by improving their geometry [25,26,27]. All these studies confirm the feasibility and accuracy of using the discrete element method to analyze the wear characteristics of mixing equipment.

However, most of these studies focus on horizontal-axis mixers, with limited analysis of the wear characteristics of planetary mixers. Most scholars focus on analyzing the impact of mixer structure on wear but often fail to fully consider the role of concrete materials in blade wear characteristics. In actual concrete production processes, repeated rolling friction and collision impacts occur between concrete particles and blades, which increases blade wear. Therefore, analyzing the impact of concrete materials on mixer wear is essential.

To address the aforementioned issues, this paper establishes a numerical simulation method for studying the wear characteristics of vertical-axis planetary mixer blades. First, simplified models of the mixer and four common aggregate particle shapes were created using SolidWorks 2018 software and subsequently imported into the EDEM 2022 software for simulation. This study analyzed the effects of mixing speed, blade angle, aggregate percentage, and aggregate shape on blade wear by considering both blade structure and material properties. This study aims to provide theoretical support for understanding the wear characteristics of mixer blades and for optimizing and improving mixer blade design.

## 2. Models and Methods

### 2.1. Mixer Model

**Figure 1:**
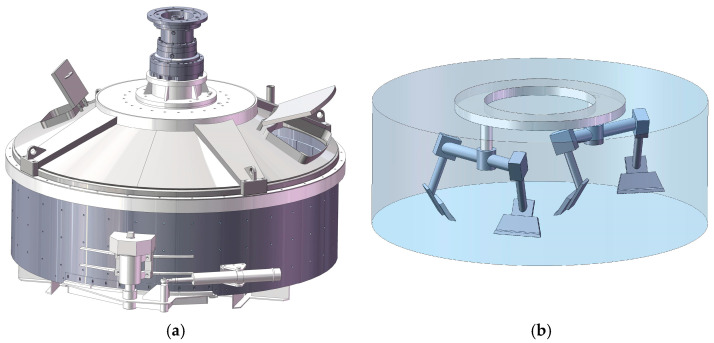
Mixer model: (**a**) three-dimensional overall modeling; (**b**) simplified model.

**Figure 2:**
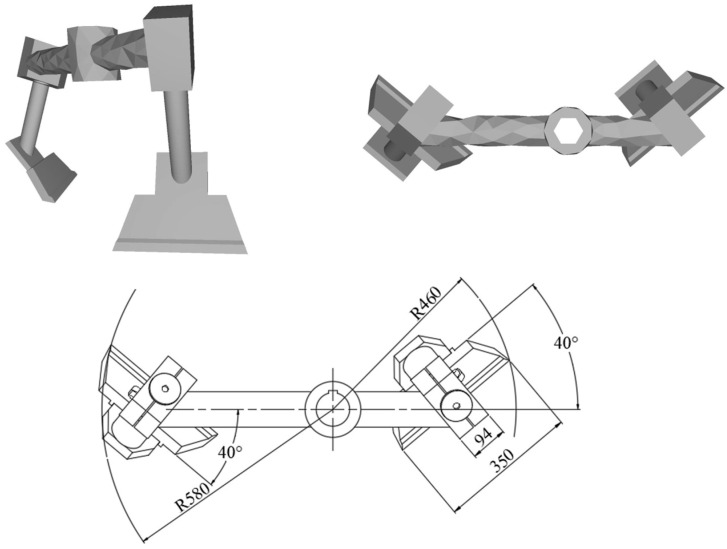
Mixing blade model and related dimensional parameters.

To perform simulation calculations, the mixer model must be simplified appropriately. In this study, the mixer model was constructed using SolidWorks 2018 3D modeling software at a 1:1 scale relative to the actual mixer and subsequently simplified. To improve the accuracy of the simulation results and reduce the consumption of computational resources, the mesh refinement of the simplified model was carried out using the meshing tool in Workbench 2022 R1 software. Figure 1 shows the model of the mixer and Figure 2 shows the blade model and its key dimensional parameters.

### 2.2. Discrete Element Contact Model

**Figure 3:**
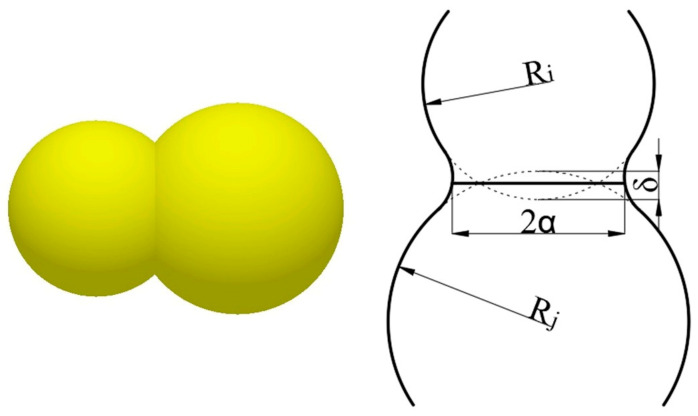
Schematic representation of interparticle bonding in the JKR contact model.

In discrete element simulation, the choice of contact model is decisive for the accuracy of the simulation results. To characterize the adhesion properties of concrete, the Hertz–Mindlin with JKR cohesive contact model [28] is used, which takes into account the effect of the adhesion between wet particles on the particle motion law, which is conducive to improving the accuracy of the simulation results. The schematic diagram of the interparticle bonding in the JKR contact theory is shown in Figure 3.

The normal contact force between the particles of this model was calculated using $F_{JKR}$:

(1) $$F_{JKR}=-4\sqrt{\pi \gamma E^{*}}\alpha^{\frac{2}{3}}+\frac{4E^{*}}{3R^{*}}\alpha^{3}$$

where $F_{JKR}$ is the *JKR* normal force (*N*), γ is the surface energy (J/m^2^), $E^{*}$ is the equivalent Young’s modulus, $R^{*}$ is the equivalent radius, and α is the tangential overlap.

The relationship between the tangential overlap amount α and the normal overlap amount δ is as follows:

(2) $$\delta =\frac{\alpha^{2}}{R^{*}}-\sqrt{4\pi \gamma \alpha /E^{*}}$$

where the equivalent Young’s modulus $E^{*}$ and the equivalent radius $R^{*}$ are defined as follows:

(3) $$E_{i}\cdot \frac{1}{E^{*}}=\frac{\left(1-{v_{i}}^{2}\right)}{E_{i}}+\frac{\left(1-{v_{j}}^{2}\right)}{E_{j}}$$

(4) $$\frac{1}{R^{*}}=\frac{1}{R_{i}}+\frac{1}{R_{j}}$$

In Equations (3) and (4), $E_{i}$, $v_{i}$, $R_{i}$ and $E_{j}$, $v_{j}$, $R_{j}$ are the Young’s modulus, Poisson’s ratio, and radius of the contact sphere, respectively.

### 2.3. Model of Concrete Aggregate

**Figure 4:**
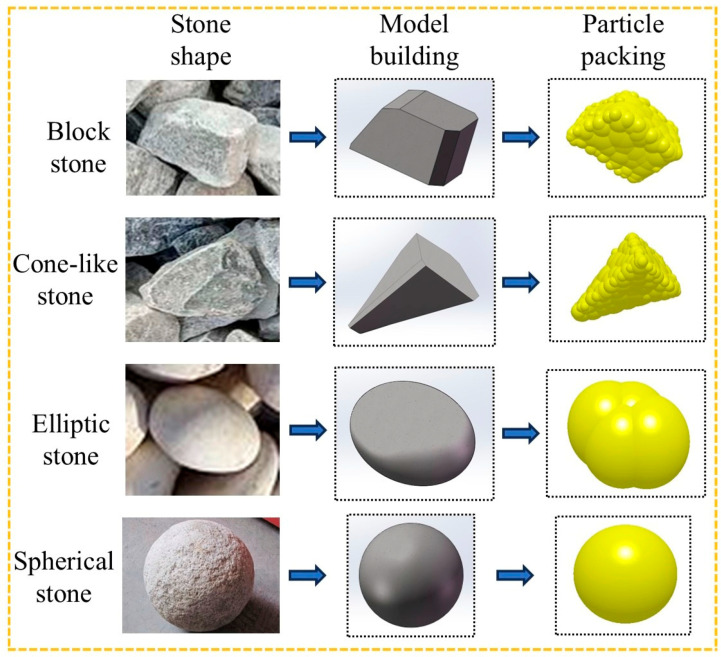
Aggregate modeling with different shapes.

Common concrete aggregates contain two categories: crushed stone and pebbles, while crushed stone contains more regular blocky stones and irregular conical stones, and pebbles contain oval stones and spherical stones, among which crushed stone is most widely used in concrete production [29,30]. Some scholars have analyzed the effect of aggregate shape on mixing efficiency [31]. Firstly, the three-dimensional models of the four types of gravel are established in SolidWorks 2018 software according to their actual shapes, ensuring that the volumes of these models are the same. Then, the models are imported into the EDEM 2022 discrete element software for particle filling, ultimately generating these four types of gravel aggregate particles. Due to the small and standardized shape of cement and sand particles, a kind of spherical cement mortar particle is established concerning the research of other scholars to facilitate the simulation calculation. The stone aggregate particle model is shown in Figure 4.

Considering the actual production materials of mixer blades, carbon steel was selected as the blade base material. Since the numerical simulation method cannot perform heat treatment or surface treatment on the material, to ensure the authenticity of the blade, we set the Poisson’s ratio, density, and shear modulus of the blade material in the simulation. These parameter settings are based on the typical values of actual carbon steel. By setting these parameters, we were able to simulate mechanical behavior similar to that of an actual carbon steel blade, ensuring the accuracy and reliability of the simulation. The material properties of the blade and particles are listed in Table 1, and the contact parameters are listed in Table 2, with surface energy parameters referenced in [25].

### 2.4. Simulation Scheme and Simulation Parameter Setting

In this study, the effect of specific parameters on the wear of mixer blades was analyzed by controlling a single-variable method. The total amount of material is 750 kg as the base material parameter for simulation analysis; mixing speed 25 rpm (autorotation), blade angle 40°, aggregate mass percentage 60%, and elliptical aggregate are taken as the base characteristic parameters. Considering the integrity of the simulation result data and the utilization of computational resources, a total simulation time of 10 s is selected. Additionally, the time step is set to 20% of the Riley time step, and the grid size is chosen to be 2.5 times the minimum particle radius (2.5 Rmin). The simulation scenarios are shown in Table 3.

## 3. Results and Discussion

### 3.1. Preliminary Simulation Analysis

**Figure 5:**
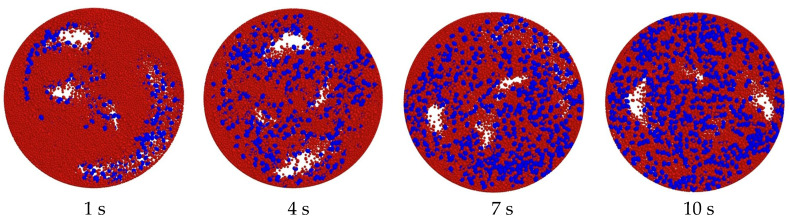
Mixing state of materials at different moments.

**Figure 6:**
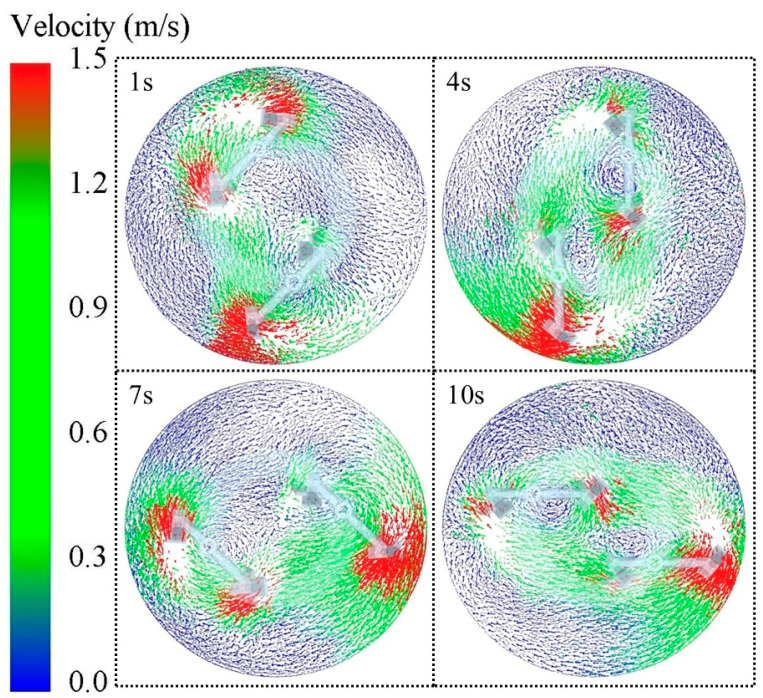
Vectors of particle velocities at different moments.

This study begins with a preliminary analysis based on the underlying characteristic parameters. In the simulation process, the aggregate (blue) is placed in the bottom layer of the mixer and the cement mortar particles (red) are placed in the top layer. Figure 5 demonstrates the material mixing state at different moments, and it can be observed that the mixing homogeneity of aggregate and mortar particles gradually increases as time advances. Figure 6 shows the vector plot of particle velocity during the material mixing process, from which it is obvious that the particle velocity reaches its maximum value near the mixing blades. This is due to the mixing effect of the blade: the blade contact area of the material particles by the blade force is the largest, with the most intense particle movement. Correspondingly, the mixing blades are subjected to a greater reaction force from the particles, which makes the blades more susceptible to impact wear and abrasive wear. Although the material moves in a clockwise direction during the mixing process and there may be areas of low mixing efficiency, the simulation results show that there are no dead zones where no mixing occurs at all.

**Figure 7:**
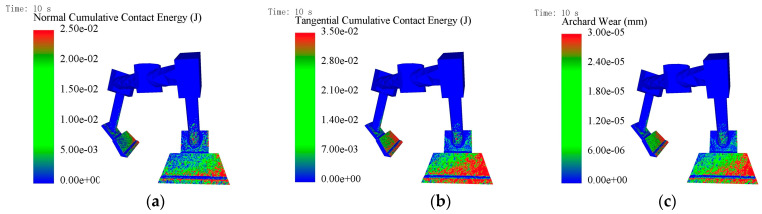
Blade simulation cloud: (**a**) average normal cumulative contact energy cloud; (**b**) average tangential cumulative contact energy cloud; (**c**) average wear depth cloud.

Figure 7a–c show the average cumulative contact energy cloud and wear depth cloud for the mixing blades, respectively. Here, the average cumulative contact energy is the total contact energy to which the blade is subjected, divided by the number of contacting particles to obtain an average value; the wear depth is expressed as an absolute value. It can be observed from the plots that the cumulative contact energy is higher in the outer edge region of the blade, and the corresponding wear is more severe, with the maximum wear location occurring in the outermost middle and lower regions of the blade, while the wear on the inner side is relatively lighter. This phenomenon can be attributed to the higher linear velocity of the blades in the outer region during the mixing process, resulting in a more violent collision with the particles, and at the same time, due to the centrifugal force, the concrete material in the outer region of the mixing drum is relatively large, and therefore comes into contact with the blades more frequently, which in turn aggravates the wear. These graphs reveal the spatial distribution law of the blade surface wear, which is consistent with the actual situation. By comparing Figure 7a,b, it can be found that the tangential cumulative contact energy exceeds the normal cumulative contact energy, which indicates that the wear of the blade in the mixing process is mainly caused by the sliding or rolling of the particles on the surface of the blade, while the wear caused by the impact of the particles on the blade is relatively small, and thus it can be deduced that the type of blade wear is abrasive wear accompanied by the impact effect.

**Figure 8:**
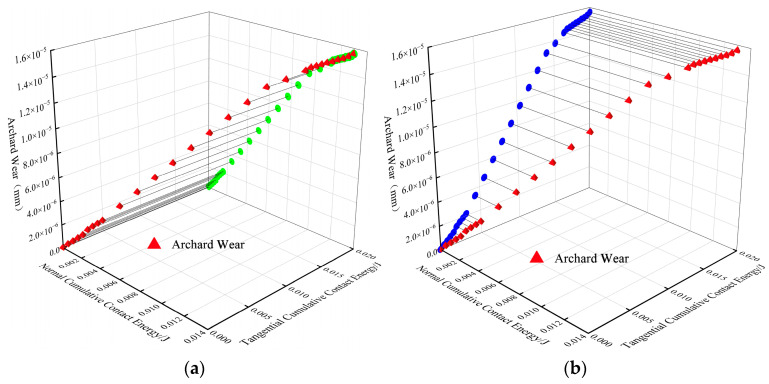
Plot of contact energy versus wear: (**a**) plot of average normal cumulative contact energy vs. wear depth; (**b**) plot of average tangential cumulative contact energy vs. wear depth.

Figure 8 demonstrates the relationship between blade cumulative contact energy and wear (the green circles indicate the relationship between normal contact energy and wear; the blue circles indicate the relationship between tangential contact energy and wear), showing that the degree of blade wear is correlated with the mean normal cumulative contact energy and the mean tangential cumulative contact energy. This study continues by analyzing the effects of different factors on the average blade wear depth, wear rate, average particle velocity, and blade-related contact data to investigate in depth the role of these factors on the wear characteristics of the blades of the mixer.

### 3.2. Effect of Different Factors on Blade Wear

#### 3.2.1. Effect of Mixing Speed on Blade Wear

**Figure 9:**
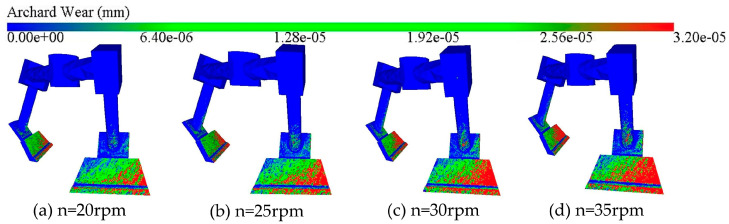
Clouds of blade wear depth at different mixing speeds.

Figure 9 is a cloud diagram of the wear depth of the mixer blade; it can be seen that the spatial distribution of the wear area of the blade under different mixing speeds is consistent with the law of the preliminary analysis, mainly distributed in the middle and lower regions of the outermost part of the blade. The faster the mixing speed, the more extensive and intensive the red area on the blade (representing the serious wear area), which indicates that the scope of blade wear expands with the increase in mixing speed, and the degree of wear is also aggravated with the increase in mixing speed.

**Figure 10:**
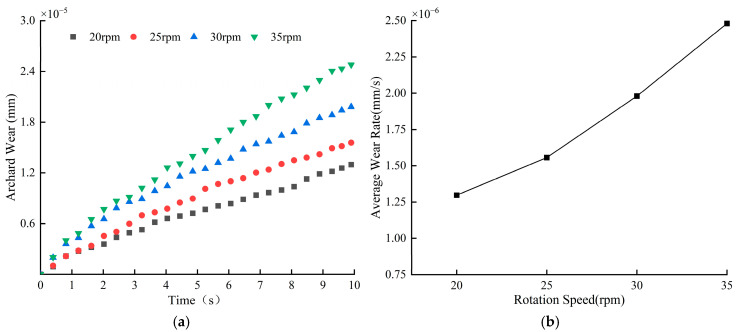
Blade wear depth and wear rate at different mixing speeds: (**a**) average blade wear depth; (**b**) blade wear rate.

Figure 10 demonstrates the average blade wear depth versus wear rate. From Figure 10a, it can be found that the blade wear depth increases linearly with time at different stirring speeds. Within 10 s of mixing, the maximum blade wear was 2.48 × 10^−5^ mm at 35 rpm, and the minimum blade wear was 1.28 × 10^−5^ mm at 20 rpm. Figure 10b shows the blade wear rate at different mixing speeds, and it can be seen that with the increase in mixing speed, the blade wear rate increases gradually. The increase in blade wear rate is more significant from 25 rpm to 35 rpm. Overall, for every 5 rpm increase in speed, the blade wear rate increases by 24.14% on average. Combined with Figure 9 and Figure 10, it can be found that the variation in blade wear under different mixing speeds is large, which indicates that the mixing speed has a significant effect on the degree of blade wear; the faster the mixing speed, the greater the depth of blade wear within the same time.

**Figure 11:**
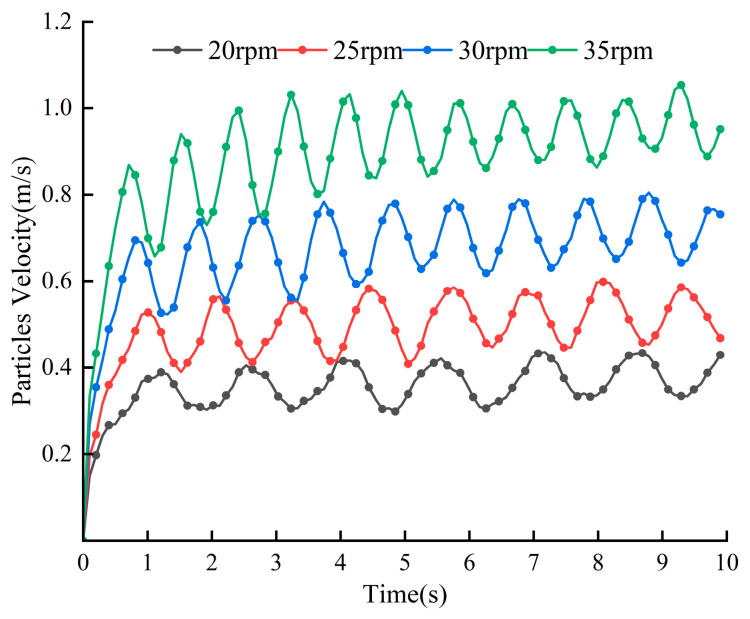
Average particle velocity at different mixing speeds.

**Figure 12:**
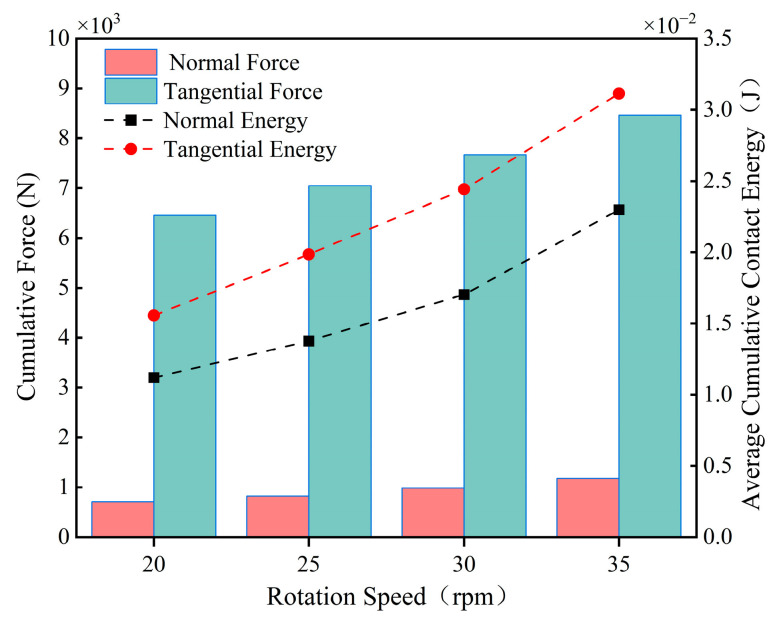
Blade cumulative contact force and average cumulative contact energy at different mixing speeds.

Figure 11 demonstrates the variation in the average velocity of the particles with time at different stirring speeds, from which it can be seen that the acceleration stage of the particles varies by 0~1 s, the velocity of the particles changes periodically by 1~10 s, and the velocity ranges of the particles at these four rotational speeds are 0.3 m/s~0.42 m/s, 0.4 m/s~0.58 m/s, 0.55 m/s~0.8 m/s, and 0.75 m/s~1.1 m/s; the overall speed of the particles is the largest and the frequency of movement is the highest when the stirring speed is 35 rpm, and the movement of the material particles is the most intense at this speed. Figure 12 shows the average cumulative contact energy of the blades under different stirring speeds about the contact force, the figure contains two components, normal and tangential. From the figure, it can be found that with the increase in rotational speed, the contact energy and contact force in both directions on the mixing blades show an obvious increasing trend; at a stirring speed of 20 rpm, the contact energy and contact force are the lowest. At this speed, the interaction between the blades and the particles is weaker, indirectly reflecting the lowest energy consumption when the two are in contact. When the stirring speed increases from 30 rpm to 35 rpm, the cumulative contact energy and contact force of the blades in both directions increases more significantly, resulting in increased energy consumption. The contact energy and contact force in the tangential direction are significantly higher than those in the normal direction at different mixing speeds.

Combined with the above data, it can be seen that the effect of mixing speed on the blade wear law is mainly to increase the contact frequency between the particles and the blade and the energy of the particles and the blade collision, which changes the cumulative contact force of the blade in both directions, thus changing the wear rate of the blade, and the mixing speed of the blade wear is more obvious.

#### 3.2.2. Effect of Blade Angle on Blade Wear

**Figure 13:**
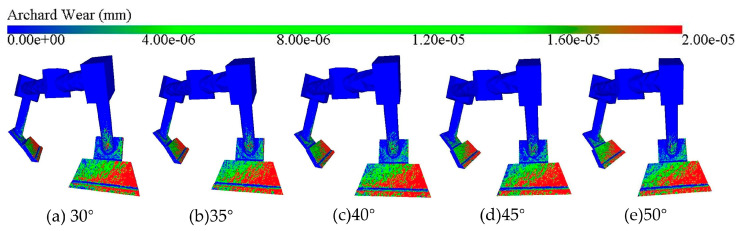
Clouds of blade wear depth at different angles.

The blade angle is a key factor affecting the mixer force, which influences the interaction between the blade and the concrete material, and thus the blade wear condition. The cloud map of blade wear depth shown in Figure 13 shows that the spatial distribution of blade wear areas is similar to that in the preliminary simulation analysis, indicating that the spatial distribution pattern of blade wear is independent of these factors, which will not be repeated in the subsequent discussion. Observation of the illustration reveals that there is no significant difference in the area and density of the red areas despite the different blade angles, which indicates that there is not much difference in blade wear at the macroscopic level.

**Figure 14:**
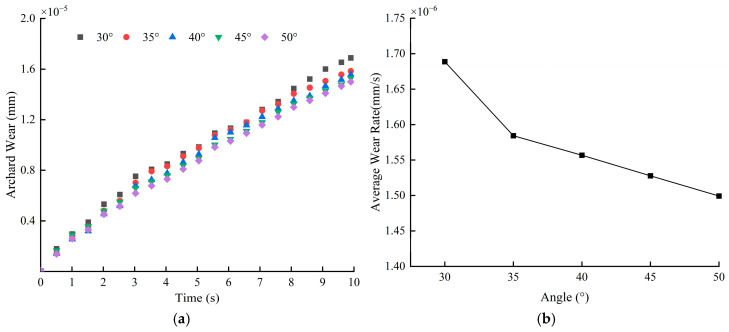
Blade wear depth and wear rate at different blade angles: (**a**) average blade wear depth; (**b**) blade wear rate.

Figure 14 shows the average wear depth and wear rate of the blade at different blade angles. From Figure 14a, it can be seen that when the blade angle is 30°, the blade wear is the most serious, with a wear depth of 1.69 × 10^−5^ mm; while when the blade angle is 50°, the wear is the lightest, with a wear depth of 1.50 × 10^−5^ mm. This indicates that the larger the blade angle, the smaller the wear depth. Although there is a difference in the wear depth at different blade angles, the difference is relatively small, indicating that the influence of blade angle on blade wear is small compared to the mixing speed. Analyzing Figure 14b, it is found that the wear rate shows a linear decrease with the increase in blade angle; in particular, when the blade angle increases from 30° to 35°, the reduction in wear rate is most significant, decreasing by 6.18%. Whenever the blade angle increases by 5°, the wear rate decreases by 2.9% on average.

**Figure 15:**
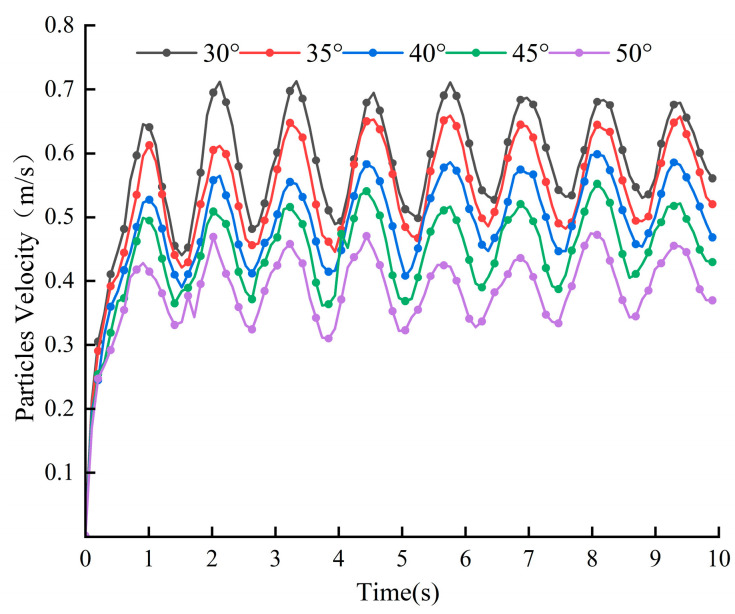
Average particle velocity at different blade angles.

**Figure 16:**
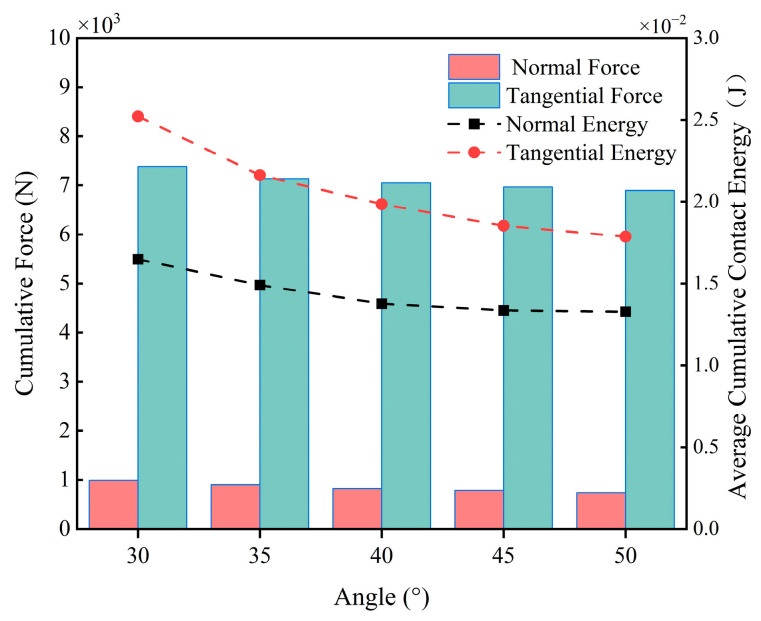
Blade cumulative contact force and average cumulative contact energy at different blade angles.

Figure 15 depicts the curve of average particle velocity with time. Data show that when the blade angle is 30° or 35°, the overall speed of particles and fluctuations range from 0.42 m/s to 0.71 m/s, indicating a more intense state of movement. Conversely, when the blade angle is 40°, 45°, or 50°, the particle speed is relatively low, with a smaller amplitude of fluctuations, and the speed range is between 0.33 m/s and 0.58 m/s, resulting in a relatively smooth state of movement. Figure 16 shows that at 30°, the cumulative contact energy and contact force in both directions reach their maximum values, and the highest energy consumption occurs at this angle. As the blade angle increases further, the contact energy and force in both directions decrease, and energy consumption gradually decreases, especially in the range from 35° to 40°, where the decrease is more pronounced. The change in the tangential direction is more pronounced than in the normal direction.

Through the analysis, it can be found that the larger the blade angle, the less significant the blade wear. The influence of mixing speed and blade angle on blade wear, as obtained through simulation analysis, is consistent with the experimental results of Petrescu et al. [10] for horizontal shaft mixers.

#### 3.2.3. Effect of Aggregate Percentage on Blade Wear

**Figure 17:**
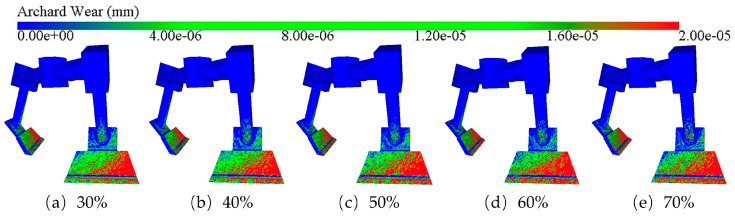
Clouds of blade wear depth at different aggregate percentages.

Figure 17 illustrates the cloud diagram of blade wear depth for different aggregate percentages. It is observed that as the aggregate percentage increases, the area of the red region indicating severe wear in the cloud diagram increases accordingly. This indicates that there is a significant difference in the macroscopic wear of the blades under different aggregate percentage conditions, thus indicating that the aggregate percentage has a significant effect on the degree of blade wear.

**Figure 18:**
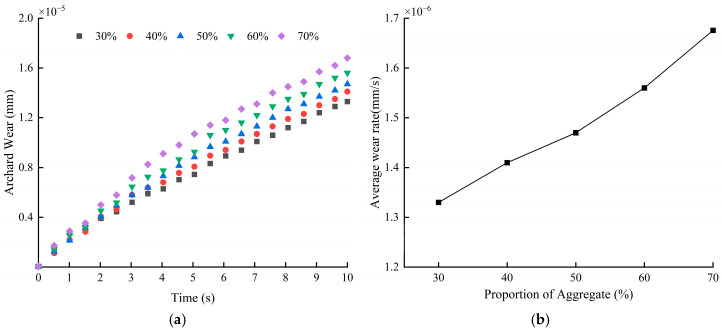
Blade wear depth and wear rate at different aggregate percentages: (**a**) average blade wear depth; (**b**) blade wear rate.

Figure 18a depicts the change rule of the average blade wear depth with time, which can be seen that it is consistent with the wear law under different rotational speeds and blade angles previously analyzed, i.e., the blade wear depth also increases linearly with time under different aggregate percentage conditions. Specifically, when the aggregate percentage is 30%, the blade wear is the lightest, and the wear depth reaches 1.33 × 10^−5^ mm, while when the aggregate percentage increases to 70%, the blade wear is the most serious, and the wear depth is 1.67 × 10^−5^ mm. In summary, the increase in aggregate percentage leads to an intensification in blade wear. From the blade wear rate graph in Figure 18b, it can be seen that with the increase in aggregate percentage, the blade wear rate shows a linear growth trend. The change in the blade wear rate is minimized in the interval where the aggregate percentage increases from 40% to 50%. Further analysis of the data in the graph reveals that whenever the aggregate mass percentage increases by 10%, the blade wear rate increases by 5.95% on average.

**Figure 19:**
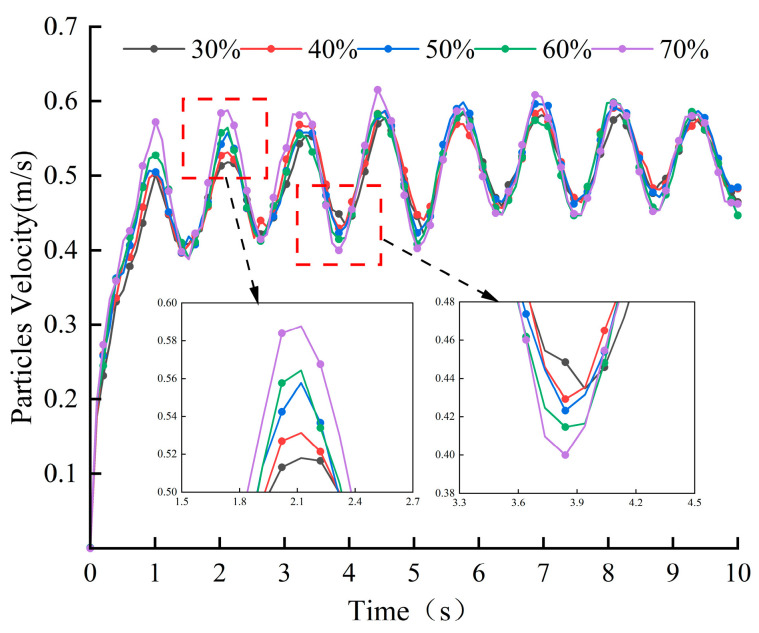
Average particle velocity at different aggregate percentages.

**Figure 20:**
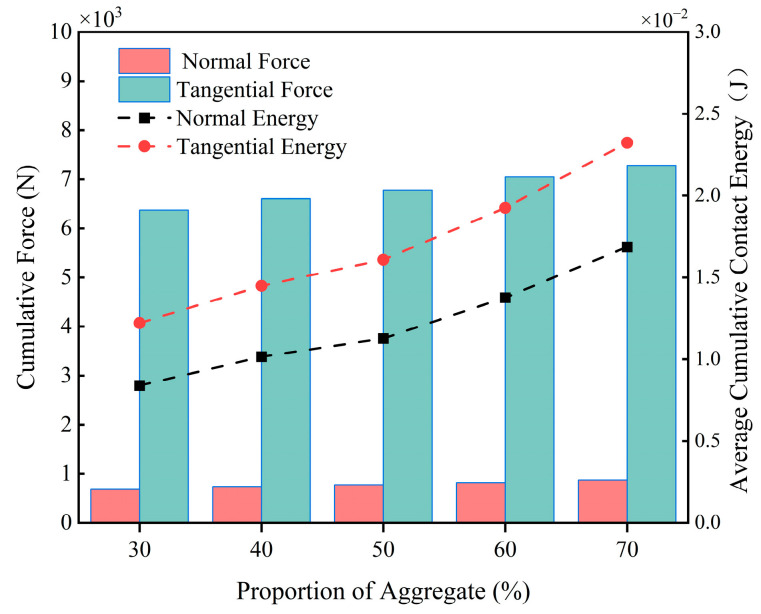
Blade cumulative contact force and average cumulative contact energy at different aggregate percentages.

Figure 19 depicts the curves of the average particle velocity with time, from which it can be seen that the particle velocity varies periodically with time and the average velocity of the particles ranges from 0.4 m/s to 0.6 m/s, and there is not much difference between the particle velocities at different aggregate percentages. By observing the two local zoomed-in areas in the figure, it can be found that the particle velocity and fluctuation amplitude gradually increase with the increase in aggregate percentage. When the aggregate percentage is 30%, the velocity and fluctuation amplitude of material particles are the smallest, while when the aggregate percentage reaches 70%, the velocity and fluctuation amplitude of material particles are the largest. Figure 20 shows that as the proportion of aggregate increases, the cumulative contact energy and contact force in both directions shows an upward trend, indicating that the energy consumption during contact gradually increases, with the trend in the tangential direction being particularly pronounced. At a 70% aggregate proportion, the contact energy and contact force in both directions reach their maximum values, and the energy consumption is highest at this proportion.

Based on the above analysis, it can be concluded that the higher the proportion of aggregate, the faster the particle velocity during the material mixing process, and the greater the fluctuation amplitude. Moreover, due to the high hardness of the aggregate, the interaction forces and energy generated during the collision between particles and blades are greater, which in turn leads to more severe wear of the blades.

#### 3.2.4. Effect of Aggregate Shape on Blade Wear

**Figure 21:**
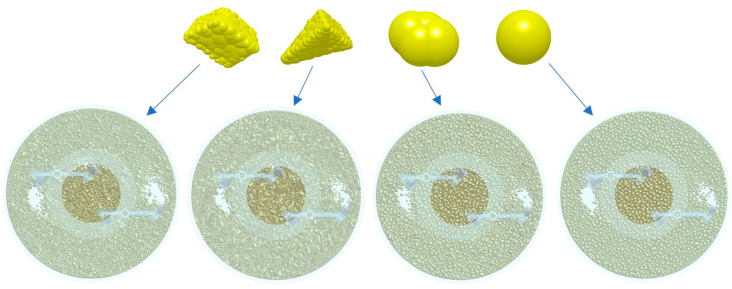
Material mixing state.

From the above analysis, we learned that aggregate particles are the main material factors causing blade wear. The effect of four common shapes of stone aggregate on blade wear is analyzed below. The concrete mix state of different aggregate shapes is shown in Figure 21.

**Figure 22:**
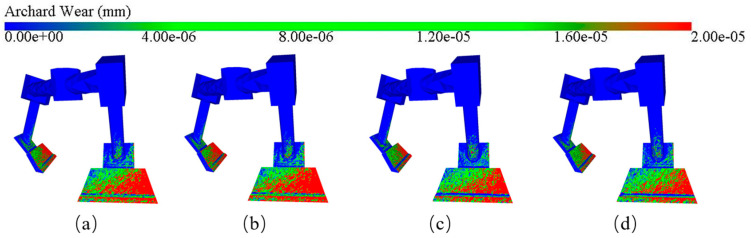
Clouds of blade wear depth at different aggregate shapes: (**a**) block aggregate; (**b**) cone-like aggregate; (**c**) elliptic aggregate; (**d**) spherical aggregate.

Figure 22 shows the cloud diagram of blade wear depth for different aggregate shapes. It can be observed from the figure that when the aggregate shape is lumpy and conical, the area of the wear region on the blade is the largest and most densely distributed, indicating that these two shapes of aggregate lead to the most serious wear of the blade. Comparatively, elliptical and spherical aggregates corresponded to smaller red areas on the blades, indicating less severe wear.

**Figure 23:**
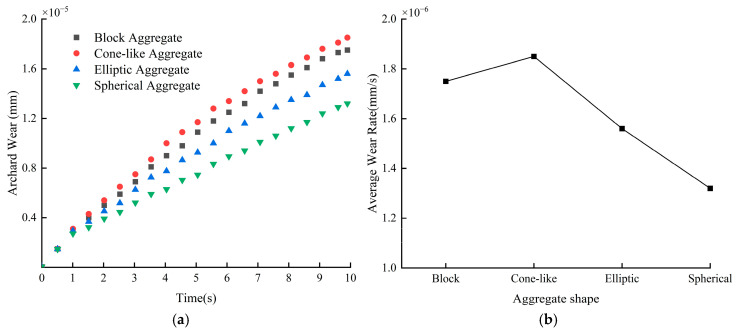
Blade wear depth and wear rate at different aggregate shapes: (**a**) average blade wear depth; (**b**) blade wear rate.

Figure 23a depicts the change rule of blade wear depth with time under different aggregate shapes. According to the data in the figure, the blade wear caused by conical aggregate is the most significant, and its wear depth reaches 1.85 × 10^−5^ mm, while the wear caused by spherical aggregate is the smallest, and the wear depth is 1.32 × 10^−5^ mm. According to Figure 23b, it can be seen that the wear rate of the blade under two kinds of crushed stone aggregate is high, the difference between the two is small, and the wear rate of the blade under the two kinds of cobblestone aggregate is small.

**Figure 24:**
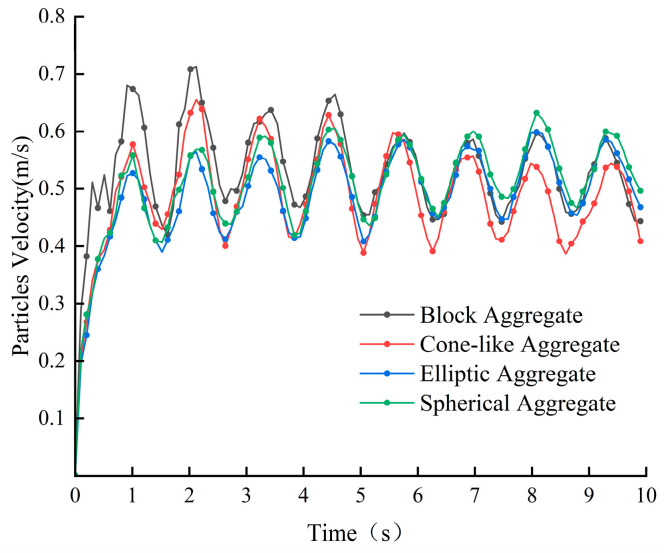
Average particle velocity at different aggregate shapes.

**Figure 25:**
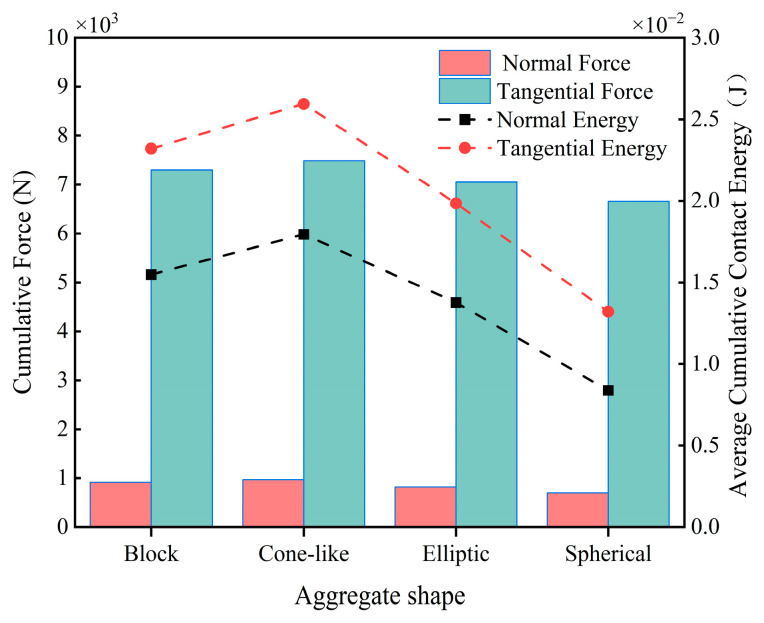
Blade cumulative contact force and average cumulative contact energy at different aggregate shapes.

As can be seen from the average particle velocity curves in Figure 24, the particle velocities under all four aggregates vary periodically with time. The particle velocities of the two types of crushed stone aggregates were overall greater at 0~5 s, with the average particle velocity under the block aggregate being the greatest, reaching a maximum of 0.72 m/s; these two particle velocities decreased overall in 5~10 s. The velocity fluctuations in the gravel aggregate particles were significantly larger than those in the pebble aggregate. The average velocity of the two pebble aggregates remained between 0.41 m/s and 0.63 m/s during the mixing process for 10 s, with no significant change. This indicates that when mixing concrete with aggregates of crushed stone, the mixing state is the most unstable, and the particle velocity varies greatly, which is more likely to harm the blades. Observation of Figure 25 shows that the conical-like aggregate produces the largest contact energy and force on the blade, resulting in the highest energy dissipation when the two collide. Conversely, the spherical aggregate produces the smallest contact energy and force on the blade, resulting in the lowest energy dissipation during collision. This is because the sharp shape of conical aggregate easily forms a localized high-stress area on the blade surface, leading to impact wear and fatigue wear, thus accelerating damage to the blade.

Combined with the above analysis, it can be seen that gravel is more likely to cause wear on the blade compared to pebbles.

## 4. Conclusions

In this study, the mixing process of concrete materials in a vertical shaft planetary mixer was simulated using the numerical simulation method. By analyzing the effects of different factors on the wear of mixer blades, the following conclusions were drawn:

(1) The type of blade wear is abrasive wear accompanied by impact. The maximum wear location occurs in the outermost middle and lower regions of the blade, which is consistent with the actual spatial distribution of wear.
(2) The blade wear depth increases linearly with the increase in mixing speed. The higher the mixing speed, the more intense the particle movement, the higher the contact frequency of particles with the blade, and the greater the cumulative contact energy and force experienced by the blade, resulting in more severe blade wear.
(3) The depth of blade wear decreases with the increase in blade angle. The larger the blade angle, the slower the particles move, the less contact energy and contact force the blade is subjected to, and the less the blade wears.
(4) The depth of blade wear increases with the increase in the proportion of stone aggregate. Stone aggregate is the main material factor causing blade wear: the larger the proportion of stone aggregate, the larger the impact load of the blade by particle collision, and the more serious the degree of blade wear.
(5) Cone-like stone aggregate has the most serious impact on blade wear, while the impact of spherical aggregate is the lightest. The gravel aggregate is more likely to form a localized high-stress region on the blade surface due to its sharp edges, thus increasing blade wear.

This study focuses on the effects of different factors on the wear of mixer blades through numerical simulation methods, but it cannot simulate the physical and chemical reactions generated during the concrete mixing process. Therefore, subsequent study will be guided by the numerical simulation results and verified with actual experimental results.

## Figures and Tables

**Table 1:** Properties of materials.

Material	Poisson’s Ratio	Density (kg/m^3^)	Shear Modulus (GPa)
Aggregate	0.35	2500	20
Mortar	0.25	2000	3
Blade	0.3	7850	70

**Table 2:** Material-to-material contact parameters.

Title 1	Coefficient of Restitution	Coefficient of Static Friction	Coefficient of Rolling Friction	Surface Energy (J/m^2^)
Aggregate–Aggregate	0.7	0.3	0.02	1
Aggregate–Mortar	0.8	0.35	0.015	6
Aggregate–Blade	0.6	0.2	0.03	0.1
Mortar–Mortar	0.7	0.3	0.01	3
Mortar–Blade	0.5	0.1	0.025	0.3

**Table 3:** Numerical simulation program.

Factor	Case	Rotation Speed (rpm)	Blade Angle (°)	Proportion of Aggregate (%)	Aggregate Type
Rotation speed	1	20	40	60	Elliptic
	2	25	40	60	Elliptic
	3	30	40	60	Elliptic
	4	35	40	60	Elliptic
Blade angle	1	25	30	60	Elliptic
	2	25	35	60	Elliptic
	3	25	40	60	Elliptic
	4	25	45	60	Elliptic
	5	25	50	60	Elliptic
Proportion of aggregate	1	25	40	30	Elliptic
	2	25	40	40	Elliptic
	3	25	40	50	Elliptic
	4	25	40	60	Elliptic
	5	25	40	70	Elliptic
Aggregate type	1	25	40	60	Block
	2	25	40	60	Cone-like
	3	25	40	60	Elliptic
	4	25	40	60	Spherical

## Data Availability

The original contributions presented in this study are included in the article. Further inquiries can be directed to the first author.
